# Fitness outcomes from a randomised controlled trial of exercise training for men with prostate cancer: the ENGAGE study

**DOI:** 10.1007/s11764-016-0543-6

**Published:** 2016-04-20

**Authors:** Cadeyrn J. Gaskin, Steve F. Fraser, Patrick J. Owen, Melinda Craike, Liliana Orellana, Patricia M. Livingston

**Affiliations:** 1 Faculty of Health, Deakin University, Locked Bag 20001, Geelong, Victoria 3220 Australia; 2 Institute for Physical Activity and Nutrition Research, School of Exercise and Nutrition Sciences, Faculty of Health, Deakin University, Geelong, Australia; 3 Institute of Sport, Exercise and Active Living, College of Sport and Exercise Science, Victoria University, Melbourne, Australia; 4 Biostatistics Unit, Faculty of Health, Deakin University, Geelong, Australia

**Keywords:** Prostate cancer, Androgen deprivation therapy, Aerobic exercise training, Resistance exercise training, Fitness, Physical function

## Abstract

**Purpose:**

The main purpose of this study was to investigate the effects of a 12-week, clinician-referred, community-based exercise training program with supervised and unsupervised sessions for men with prostate cancer. The secondary purpose was to determine whether androgen deprivation therapy (ADT) modified responses to exercise training.

**Methods:**

Secondary analysis was undertaken on data from a multicentre cluster randomised controlled trial in which 15 clinicians were randomly assigned to refer eligible patients to an exercise training intervention (*n* = 8) or to provide usual care (*n* = 7). Data from 119 patients (intervention *n* = 53, control *n* = 66) were available for this analysis. Outcome measures included fitness and physical function, anthropometrics, resting heart rate, and blood pressure.

**Results:**

Compared to the control condition, men in the intervention significantly improved their 6-min walk distance (*M*_diff_ = 49.98 m, *p*_adj_ = 0.001), leg strength (*M*_diff_ = 21.82 kg, *p*_adj_ = 0.001), chest strength (*M*_diff_ = 6.91 kg, *p*_adj_ = 0.001), 30-s sit-to-stand result (*M*_diff_ = 3.38 reps, *p*_adj_ = 0.001), and reach distance (*M*_diff_ = 4.8 cm, *p*_adj_ = 0.024). A significant difference (unadjusted for multiplicity) in favour of men in the intervention was also found for resting heart rate (*M*_diff_ = −3.76 beats/min, *p* = 0.034). ADT did not modify responses to exercise training.

**Conclusions:**

Men with prostate cancer who act upon clinician referrals to community-based exercise training programs can improve their strength, physical functioning, and, potentially, cardiovascular health, irrespective of whether or not they are treated with ADT.

**Implications for Cancer Survivors:**

Clinicians should inform men with prostate cancer about the benefits of exercise and refer them to appropriately qualified exercise practitioners and suitable community-based programs.

**Trial registration:**

Australia and New Zealand Clinical Trials Register (ANZCTR): ACTRN12610000609055

## Introduction

Prostate cancer is the most frequently diagnosed cancer among men in more developed regions of the world [1], with the trend in 5-year relative survival rates approaching 100 % in some counties [2–4]. Men with prostate cancer typically experience adverse effects associated with the disease and its treatment, however, including urinary leakage, bowel urgency, erectile dysfunction [5], fatigue [6], anxiety and depression [7], and diminished health-related quality of life [8]. In addition, those treated with androgen deprivation therapy (ADT) are at increased risk of bone fracture, muscle mass and strength loss, diabetes, obesity, and cardiovascular-related mortality [9, 10]. With this profile of adverse effects, counselling men with prostate cancer to engage in exercise training, in combination with other health promoting activities, has been recommended [11].

Exercise training can improve the health and well-being of men with prostate cancer, including aerobic fitness, muscular performance (endurance and strength), fatigue, flexibility, body composition (lean body mass), blood lipids, and quality of life [12–14]. The evidence is less clear, however, on the effects of exercise training on other parameters, such as blood pressure [15–17]. In addition, greater clarity is needed with regard to whether responses to exercise training differ between men with prostate cancer receiving ADT and those not receiving this treatment. Studies conducted thus far have shown minimal differences between these two cohorts [18, 19]. Given the potential of exercise training to reduce the severity of many of the adverse effects of ADT [10], identifying differences between these two cohorts in their responses to exercise training would inform the design of more effective programs.

One of the features of previous studies involving exercise training and prostate cancer is that trials have been conducted using supervised (i.e. in the presence of a therapist or exercise physiologist) or unsupervised (e.g. home-based without a therapist or exercise physiologist) programs [13, 14]. Demonstrating the effectiveness of a combined program has value, as men with prostate cancer are likely to have opportunities to undertake supervised and unsupervised exercise training in their communities (e.g. gyms that offer supervised classes and unstructured, self-initiated training). There seems to be merit in continuing this line of enquiry through designing interventions with features that replicate circumstances that men with prostate cancer are likely to experience.

With exercise training emerging as a key intervention for improving the lives of men with prostate cancer [20], we sought to clarify its effects further in community settings. We report findings from a cluster randomised controlled trial of exercise training for men with prostate cancer. This trial had several features that enhanced the ecological validity of the intervention: (a) clinicians referred men with prostate cancer to the exercise training program, (b) the program involved both supervised training and unsupervised home-based sessions, and (c) the supervised sessions were conducted in community gyms. The primary aim of the study was to investigate the effects of the 12-week exercise training program on fitness and physical function, anthropometric measurements, resting heart rate, and blood pressure. The secondary aim was to determine whether the effects of the program, in terms of exercise outcomes, were different for men who received and those who had not received ADT.

## Method

### Design

This study represents planned secondary analysis of data from a multicentre, cluster randomised controlled trial to determine the efficacy of a clinician referral and 12-week exercise training program for improving exercise levels and quality of life for men with prostate cancer. A detailed description of the study methods is available from the published protocol [21] and the main outcomes have been published [22]. In brief, 15 clinicians were randomised to intervention and control conditions. During consultations, clinicians in both conditions determined each patient’s eligibility to participate in the trial. Using short pre-prepared scripts, clinicians recommended eligible patients participate in the trial and advised ineligible patients not to participate [21]. Clinicians in the intervention condition (*n* = 8) referred eligible patients to an exercise training program involving two supervised sessions in community gyms and one unsupervised home-based session per week. Clinicians referred patients using a standardised referral slip, which reiterated the recommendation to participate in the exercise training program. Clinicians in the control condition (*n* = 7) recommended eligible patients participate in the trial and provided usual care regarding advice on exercise training (typically, limited amounts of information are provided). Clinician adherence to the referral process was monitored weekly with emails and personal contact with clinicians at the clinics, checking that they were following the protocol and answering any queries that they may have had. The patients in both conditions completed measures associated with the primary outcomes of the main study [22], and were also invited to take part in measurements of their fitness and physical function, anthropometrics, resting heart rate, and blood pressure. Assessments were conducted before the exercise training program begun (baseline) and following the program (12 weeks).

The protocol for this study received approval from the human research ethics committee of the host university and those of the healthcare services involved with participant recruitment. Each participant provided written informed consent.

### Participants

The 15 clinicians involved in this study practised at urology and radiation oncology outpatient clinics across three major public health services and four private clinics in Melbourne, Australia. Their patients (*n* = 741) were screened to determine their eligibility for this study (see Table 1).

**Table 1:** Study inclusion and exclusion criteria

Inclusion criteria
Diagnosed with stage I, II, or III prostate cancer Completed active treatment for prostate cancer within the previous 3–12 months (patients on hormone treatment were eligible to participate) The ability to complete surveys in the English language
Exclusion criteria
Any musculoskeletal, cardiovascular, or neurological disorders that could limit exercising

Sample size calculations were based on the primary outcome of the main study (self-reported involvement in moderate and strenuous physical activity) [21, 22], rather than the secondary analysis reported here.

### Exercise training intervention

The 12-week exercise training program involved each participant undertaking two supervised, 50-min sessions at their local community gym and one other home-based session per week. Under the supervision of accredited exercise physiologists (tertiary-educated exercise professionals), postgraduate exercise physiology students from two universities (in training to become accredited exercise physiologists) supervised participants during their gym sessions and provided advice and written instruction for their home-based sessions. The exercise training programs were based on exercise training guidelines for cancer survivors from the American College of Sports Medicine [23] and Exercise and Sport Science Australia [24].

The supervising accredited exercise physiologists have extensive training and experience working with clinical populations, including those with cardiovascular disease, diabetes, cancer, arthritis, and chronic kidney disease. The Masters-level students, as part of their regular coursework, received education on exercise prescription for people with cancer, as well as on working with various comorbidities that may be present in this clinical population. Prior to training participants in this study, students also received an induction from the supervising accredited exercise physiologists, during which prescription guidelines and training expectations for men with prostate cancer were discussed.

Supervised sessions were performed at community gyms (six YMCA centres and one university-based gym) across Melbourne. The YMCA centres and university-based gym represent accessible and affordable community gyms. For convenience, participants trained at the community gyms located closest to them. Each supervised session included 20–30 min of aerobic training, prescribed at 40–70 % of maximum heart rate, and 4–6 upper and lower body resistance training exercises, prescribed as two sets of 8–12 repetitions. To achieve consistency in exercise prescription, each participant’s program included a 90° leg press, seated chest press, and seated row. The home-based exercise training programs included both aerobic and resistance exercises, with body weight and resistance-band exercises prescribed to increase the range of exercises participants could perform independently at home. Programs were frequently modified and safely progressed to ensure continual fitness gains.

### Outcome measures

#### Fitness and physical function

Participants completed five functional exercise tests that are common and safe for cancer survivors [25]. Aerobic fitness was assessed using the 6-min walk test [26]. Upper and lower body maximal strength was measured using a one-repetition maximum protocol [25] with the 90° leg press and seated chest press. Lower body muscular endurance was assessed using the 30-s sit-to-stand test [27]. Balance was assessed using the functional reach test [28].

#### Anthropometrics

Height and body mass were measured using a portable stadiometer (220, Seca, Hamburg, Germany) and portable scales (UC-321, A&D, Tokyo, Japan), respectively. Body mass index (BMI) was then calculated. Girth measurements of the chest, waist, hips, upper arm, and mid-thigh were taken using standard techniques [29].

#### Resting heart rate and blood pressure

Assessment of resting heart rate (Electro N2965, Polar, Kempele, Finland) and blood pressure (Gamma 4.0 sphygmomanometer, Heine, Herrsching, Germany) were obtained manually. Both measures were taken after each participant had been seated for 10 min.

### Statistical analyses

Data were analysed using SAS software, version 9.3 (SAS Institute, Cary, NC). The baseline characteristics of participants in the intervention and control conditions were compared using chi-squared and Fisher’s exact tests for categorical variables (relationship status, highest level of education, stage of disease, treatment regime, ADT, and health service type) and linear mixed models (LMM) with intervention as a fixed effect and clinician as a random effect for continuous variables (age, weeks since active treatment, fitness and physical function, anthropometrics, resting heart rate, and blood pressure). All LMM analyses conducted in this study included clinician as a random effect to account for the clustering due to randomisation at the clinician level.

The effect of the intervention on changes in fitness and physical function, anthropometrics, resting heart rate, and blood pressure between baseline and end of follow-up was determined using LMM with clinician as a random effect. For this analysis, missing data at 12 weeks were imputed using the Markov chain Monte Carlo algorithm with 20 imputations [30].

To evaluate effect modification of the intervention due to ADT, LMM were fitted with intervention, ADT, and the interaction term, intervention × ADT, as fixed effects and clinician as a random effect.

Due to the effect of multiple comparisons on experiment-wise error (multiplicity), adjusted *p* values were calculated for the 16 tests included in the primary analysis. Both adjusted and unadjusted *p* values are reported for these tests. Adjusted *p* values were computed using Hommel’s [31] procedure, which has been shown to have superior statistical power to the original Bonferroni procedure and similar power to other alternatives [32].

## Results

### Baseline participant characteristics, attrition, compliance with the intervention, and safety

Of those screened, 147 patients were eligible and agreed to participate in the main study, with 54 referred from clinicians randomised to the intervention condition and 93 referred from clinicians in the control condition [22]. Of these 147 men, 119 participants (intervention *n* = 53, control *n* = 66) provided data for this secondary analysis. One man withdrew from the intervention condition before baseline data were collected for this secondary analysis, and 27 men from the control condition gave their consent to participate in the main study on the proviso that they did not have to provide physiological data for this secondary analysis. Compared to men who did not provide physiological data, those who did participate in data collection for this secondary analysis were 4.2 years older (*p* = 0.036) and had undergone different treatment regimens (more likely to have had surgery and radiotherapy [24.2 vs 0.0 %] or radiotherapy and ADT [12.1 vs 0.0 %], and less likely to have had surgery only [43.9 vs 63.0 %], radiotherapy only [16.7 vs 18.5 %], surgery, radiotherapy, and ADT [3.0 vs 11.1 %], or surgery and ADT [0.0 vs 7.4 %], *p* = 0.0006). In addition, 4.5 weeks more had elapsed since active treatment for the men who participated in the data collection (*p* = 0.032).

No significant differences between the intervention and control conditions were observed for any participant characteristic (see Table 2). Of the 119 participants, 11 men (intervention *n* = 6, control *n* = 5) withdrew or were lost to follow-up (refer to main study paper for details [22]). In comparison to those who underwent assessment at 12 weeks, those who did not attend this assessment had larger chests and waists, as well as higher diastolic blood pressure at baseline (*p* < 0.05 for all). No other statistically significant differences were found. Comparisons of the non-attendees from the intervention (*n* = 6) and control (*n* = 5) conditions yielded no statistically significant differences (the power to detect such differences was low, however).

**Table 2:** Baseline participant characteristics

Characteristics	Intervention (*n* = 53)	Control (*n* = 66)	*p* ^a^
	Mean ± SD or *n* (%)	Mean ± SD or *n* (%)	
Demographic characteristics			
Age (years)	66.8 ± 8.3	66.0 ± 8.1	0.276
Relationship status			0.809
Married	41 (78.8)	47 (73.4)	
De facto or living with partner	3 (5.8)	6 (9.4)	
Separated, divorced, widowed, or not living with partner	8 (15.4)	11 (17.2)	
Highest level of education			0.974
Primary school	3 (5.8)	4 (6.3)	
Secondary school	15 (28.8)	17 (27.0)	
Certificate or diploma	18 (34.6)	24 (38.1)	
University degree	16 (30.8)	18 (28.6)	
Clinical characteristics			
Stage of disease			0.075
Stage I	19 (46.3)	22 (36.7)	
Stage II	11 (26.8)	29 (48.3)	
Stage III	11 (26.8)	9 (15.0)	
Treatment regime			0.347
Surgery only	18 (34.0)	29 (43.9)	
Radiotherapy only	5 (9.4)	11 (16.7)	
Surgery and radiotherapy	15 (28.3)	16 (24.2)	
Radiotherapy and androgen deprivation therapy	12 (22.6)	8 (12.1)	
Surgery, radiotherapy, and androgen deprivation therapy	3 (5.7)	2 (3.0)	
Androgen deprivation therapy			
No	38 (71.7)	56 (84.8)	0.112
Yes	15 (28.3)	10 (15.2)	
Weeks since active treatment	25.3 ± 11.4	26.5 ± 9.4	0.586
Health service type			
Public	39 (73.6)	50 (75.8)	0.834
Private	14 (26.4)	16 (24.2)	
Physical function			
6-min walk distance (m)	543.7 ± 81.8	551.7 ± 67.9	0.793
Leg—1 repetition maximum (kg)	126.1 ± 46.0	136.8 ± 42.7	0.181
Chest—1 repetition maximum (kg)	60.5 ± 22.8	60.0 ± 21.6	0.540
30-s sit-to-stand (n)	14.2 ± 3.6	14.8 ± 4.1	0.395
Reach distance (cm)	36.4 ± 5.8	38.2 ± 7.8	0.331
Anthropometrics			
Body mass index (kg/m^2^)	28.5 ± 3.7	28.9 ± 4.0	0.510
Chest circumference (cm)	101.9 ± 7.7	102.4 ± 8.3	1.000
Waist circumference (cm)	97.7 ± 9.9	98.3 ± 10.6	0.693
Hip circumference (cm)	102.2 ± 6.5	103.1 ± 7.7	0.671
Right thigh circumference (cm)	47.9 ± 4.4	49.0 ± 4.7	0.365
Left thigh circumference (cm)	47.9 ± 4.2	48.6 ± 4.5	0.490
Right arm circumference (cm)	30.5 ± 2.9	30.8 ± 3.1	0.723
Left arm circumference (cm)	30.1 ± 3.2	30.5 ± 3.1	0.583
Resting heart rate and blood pressure			
Resting heart rate (beats/min)	71.2 ± 10.9	72.0 ± 13.3	0.444
Systolic blood pressure (mmHg)	134.6 ± 11.5	136.6 (11.9)	0.942
Diastolic blood pressure (mmHg)	83.1 ± 8.5	84.5 ± 8.2	0.448

^a^Fisher’s exact test for categorical variables, linear mixed model including intervention as fixed effect and clinician as random effect for continuous variables. No adjustments were made for multiplicity

At baseline, compared with men not receiving ADT, those receiving ADT were older (*M*_diff_ = 7.3 years, 95 % CI [3.4, 11.2], *p* < 0.0001), had higher systolic blood pressure (*M*_diff_ = 8.4 mmHg, 95 % CI [1.6, 15.2], *p* = 0.016), walked less distance over 6 min (*M*_diff_ = −44.5 m, 95 % CI [−83.8, −5.2], *p* = 0.027), and had lower chest strength (*M*_diff_ = −13.6 kg, 95 % CI [−25.2, −2.0], *p* = 0.022) and leg strength (*M*_diff_ = −43.4 kg, 95 % CI [−68.1, −18.8], *p* = 0.0007). In addition, for men receiving ADT, more time had elapsed since active treatment (*M*_diff_ = 5.9 weeks, 95 % CI [0.5, 11.3], *p* = 0.034).

As has been reported previously, 85 % of the men in the intervention condition undertook at least 18 of the 24 supervised sessions in their local community gyms and, of those who completed their exercise diaries (74 %), 81 % completed at least 9 of 12 home-based sessions [22].

Adverse events (musculoskeletal injuries) were reported for two participants. One man in the intervention condition aggravated a previous rotator cuff injury (left shoulder, grade I strain) during exercise training. The other man, in the control condition, aggravated a previous meniscus injury (right knee, inflammation) during baseline testing.

### Fitness and physical function

Sizable, statistically significant differences between conditions were observed, in favour of men in the intervention condition for all measures of physical functioning (Table 3).

**Table 3:** Changes in fitness and physical function, anthropometrics, resting heart rate, and blood pressure, and from baseline to 12 weeks: comparison between conditions

Variable	Mean change (95 % CI)		Mean intervention effect (95 % CI)		
	Intervention (*n* = 53)	Control (*n* = 66)	Intervention—control	*p* ^a^	*p*_adj_^b^
Fitness and physical function					
6-min walk distance (m)	64.87 (49.76, 79.99)	14.89 (0.47, 29.31)	49.98 (28.54, 71.42)	<0.0001	0.001
Leg—1 repetition maximum (kg)	28.54 (21.54, 35.54)	6.72 (−0.06, 13.49)	21.82 (12.15, 31.49)	<0.0001	0.001
Chest—1 repetition maximum (kg)	10.05 (7.30, 12.80)	3.14 (0.59, 5.69)	6.91 (3.31, 10.51)	<0.0001	0.001
30-s sit-to-stand (reps)	3.89 (2.80, 4.99)	0.51 (−0.54, 1.56)	3.38 (1.87, 4.89)	<0.0001	0.001
Reach distance (cm)	3.17 (0.92, 5.41)	−1.63 (−3.71, 0.45)	4.80 (1.77, 7.82)	0.002	0.024
Anthropometrics					
Body mass index (kg/m^2^)	−0.02 (−0.24, 0.20)	0.05 (−0.15, 0.24)	−0.07 (−0.36, 0.23)	0.654	0.788
Chest circumference (cm)	−0.75 (−1.66, 0.16)	−0.01 (−0.82, 0.79)	−0.74 (−1.96, 0.49)	0.238	0.788
Waist circumference (cm)	−1.06 (−2.21, 0.10)	0.06 (−0.98, 1.09)	−1.11 (−2.68, 0.46)	0.165	0.788
Hip circumference (cm)	−1.40 (−2.35, −0.46)	0.25 (−0.61, 1.11)	−1.65 (−2.95, −0.35)	0.013	0.143
Right thigh circumference (cm)	0.55 (−0.15, 1.25)	0.24 (−0.42, 0.90)	0.31 (−0.65, 1.27)	0.526	0.728
Left thigh circumference (cm)	0.66 (−0.03, 1.34)	0.35 (−0.27, 0.97)	0.31 (−0.64, 1.25)	0.523	0.788
Right arm circumference (cm)	−0.39 (−0.99, 0.21)	−0.28 (−0.81, 0.25)	−0.11 (−0.92, 0.70)	0.788	0.788
Left arm circumference (cm)	−0.34 (−0.92, 0.25)	−0.20 (−0.72, 0.32)	−0.14 (−0.93, 0.65)	0.733	0.788
Resting heart rate and blood pressure					
Resting heart rate (beats/min)	−4.21 (−6.76, −1.66)	−0.45 (−2.80, 1.90)	−3.76 (−7.23, −0.28)	0.034	0.306
Systolic blood pressure (mmHg)	−2.04 (−4.86, 0.79)	1.78 (−0.71, 4.28)	−3.82 (−7.65, 0.02)	0.051	0.459
Diastolic blood pressure (mmHg)	−1.31 (−3.52, 0.90)	0.72 (−1.25, 2.70)	−2.03 (−5.00, 0.93)	0.179	0.788

^a^ *p* values are unadjusted for multiplicity

^b^ *p* values are adjusted for multiplicity using Hommel’s [31] procedure

Examinations of the possible modifying effect of ADT treatment on the intervention effect revealed minimal, non-significant differences between men receiving ADT and those not receiving this treatment. The mean intervention effects were similar for 6-min walk distance (ADT 59.7 m, 95 % CI [11.8, 107.5]; non-ADT 47.5 m, 95 % CI [21.8, 73.2]; *p* = 0.673), leg strength (ADT 13.7 kg, 95 % CI [−8.4, 35.8]; non-ADT 25.2 kg, 95 % CI [13.9, 36.5]; *p* = 0.370), chest strength (ADT 8.5 kg, 95 % CI [−0.7, 17.7]; non-ADT 7.1 kg, 95 % CI [2.7, 11.4]; *p* = 0.788), 30-s sit-to-stand result (ADT 4.0 reps, 95 % CI [0.4, 7.6]; non-ADT 3.4 reps, 95 % CI [1.5, 5.2]; *p* = 0.778), and reach distance (ADT 7.1 cm, 95 % CI [−0.1, 14.4]; non-ADT 4.1 cm, 95 % CI [0.5, 7.7]; *p* = 0.487).

### Anthropometrics

Differences between conditions were minimal and none were statistically significant (Table 3). Changes over time were also minimal, with the only statistically significant result (unadjusted *p* only) being a 1.65-cm mean average reduction in hip circumference in favour of the men in the intervention condition.

### Resting heart rate and blood pressure

The resting heart rates of the men in the intervention condition decreased by 3.76 beats/min more than those of men in the control condition (see Table 3).

## Discussion

This trial demonstrates that the fitness and physical functioning of men with prostate cancer can be enhanced through an exercise training program with features that many men could encounter during their clinical care and within their communities. The intervention commenced with the referral of men with prostate cancer to an exercise training program—an initiative that is consistent with guidelines recommending clinicians counsel prostate cancer survivors to engage in at least 150 min of physical activity per week [11]. At present, no direct formalised pathway exists between oncology clinicians and allied health providers in Australia. People with chronic conditions and complex care needs (including people with cancer), however, may each be eligible for Medicare rebates for up to five allied health consultations per calendar year (including with accredited exercise physiologists [33]) through referrals from general practitioners. Enabling direct referrals between oncology clinicians and allied health professionals, such as exercise physiologists, would facilitate earlier intervention and improve access to exercise training programs for men with prostate cancer. The combination of supervised and unsupervised sessions also matches the reality that most men will be able to access practical support for their physical activity participation from their local communities. Community gyms (and many commercial ones), for example, typically assist members through writing exercise programs for them, providing periodic program reviews, having staff available to demonstrate correct exercise techniques, and running group exercise classes. In addition, YMCA Australia recently announced a commitment to employing accredited exercise physiologists in over 30 facilities by the end of 2018. This development will increase the accessibility of quality support and advice for men with prostate cancer who wish to follow recommendations to be physically active.

Participation in the exercise training improved the aerobic capacity of men with prostate cancer. Not only did the men improve the distances walked over 6 min, but they were also observed to have lower resting heart rates at 12 weeks, which is a common adaption of aerobic exercise training [34]. For the 6-min walk distance, the improvements (ADT 59.7 m, non-ADT 47.5 m) were in excess of the recommended criterion for meaningful change in older adults (20 m) and consistent with the criterion for substantial meaningful change (50 m) [35]. Given that the participants were already quite physically active at baseline [22] and typically did not attend all the scheduled sessions, these results may be particularly impressive. With regard to the baseline scores for the 6-min walk, for example, the men in this study covered more distance than men with prostate cancer in other studies [36, 37].

The men who participated in the exercise training increased their upper and lower body strength. Given that few randomised controlled trials focusing on these outcomes included men not treated with ADT [38], these observations are an important contribution to the literature. Developing and maintaining muscle strength is imperative, because low muscle strength is an independent predictor of impaired mobility [39] and all-cause mortality [40] in older adults. Consistent with current guidelines [11], clinicians should counsel men with prostate cancer to include resistance exercises as part of their training programs.

Encouraging men with prostate cancer to incorporate resistance exercises as part of their training also provides opportunities for their strength to be monitored (e.g. by exercise physiologists or personal trainers), and for this information to be shared with clinicians. Muscle strength is a better predictor of morbidity and all-cause mortality than muscle mass [39, 40], and the assessment of muscular strength is more feasible than common measures of muscle mass (e.g. via dual-energy X-ray absorptiometry). The assessment of muscular strength could be a valuable outcome from a referred exercise training program, because it could be used for risk stratification and further clinical interpretations.

The effect of the intervention on systolic blood pressure was less pronounced and not statistically significant, but is a finding that warrants further investigation. Previous research on men with prostate cancer has generally shown exercise to have no significant effect on systolic blood pressure [15–17, 41]. Control condition contamination, however, has been cited as a possible reason for the non-significant findings in studies of exercise and prostate cancer [41]. The magnitude of the intervention effect (−3.82 mmHg) is slightly higher than reductions achieved for adults without cardiovascular or other diseases who participate in dynamic resistance training (−1.8 mmHg) [42]. One reason for the muted response may be that the resting systolic blood pressure was not high at baseline (intervention *M* = 134.6, SD = 11.5, control *M* = 136.6, SD = 11.9), with some evidence suggesting that, for people aged over 50, systolic blood pressure <140 mmHg should be considered normal [43]. Although lowering systolic blood pressure when it is already <140 mmHg may have limited health benefits (compared with reducing systolic blood pressure to 140–160 or 140–149 mmHg) [44], changes of the magnitude observed in the present trial may be clinically meaningful with men who have higher systolic blood pressure. Clinicians and researchers are encouraged to continue investigations on the effect of exercise training on blood pressure in men with prostate cancer.

The detrimental effect of ADT on fitness and physical functioning observed previously [36, 45, 46] was also evident in the present study. At baseline, men receiving ADT had less strength, walked shorter distances over 6 min, and had higher systolic blood pressure than those not treated with ADT. Recent research has shown that declines in physical function with ADT do not recover over a 36-month period [36], which underscores the importance of using exercise training as a counteractive measure.

Men treated with ADT responded to the exercise training similarly to men who had not been treated with ADT. This finding adds weight to the small amount of previous research showing minimal, and statistically non-significant, differences between men receiving ADT and those not undergoing this treatment in terms of their responses to exercise training [18, 19]. Although some caution is appropriate when interpreting these findings (due to the comparatively low number of men receiving ADT recruited for the present study), it should be noted that the differences were essentially in favour of men receiving ADT.

A limitation of this trial was that men recruited were more physically active than those reported in other studies [47]. Higher physical activity levels at baseline may have attenuated the magnitude of the effects that may have otherwise been achieved through such an intervention with less active men with prostate cancer. If the men had been less active at baseline, clearer effects may have been observable with respect to resting heart rate, systolic blood pressure, and waist circumference. In addition, as has been mentioned, the comparatively small number of men receiving ADT recruited into this study was another limitation. A larger sample of men with ADT in this study would have enabled firmer conclusions to be drawn about the effects of ADT on men’s responses to exercise.

Despite these limitations, the trial demonstrated that clinician referral to a community-based exercise training intervention with supervised and unsupervised sessions can have clear benefits for men with prostate cancer, both in terms of their physical function and, possibly, their cardiovascular health. The findings also suggest that favourable outcomes were gained irrespective of whether or not men were treated with ADT. These results should encourage clinicians to inform their patients with prostate cancer about the benefits of exercise and to ensure they are able to refer them to appropriately qualified exercise practitioners or other supervised exercise training programs to develop suitable exercise regimens for these men.
